# Cardiac radioablation of incessant ventricular tachycardia in patients with terminal heart failure under permanent left ventricular assist device therapy—description of two cases

**DOI:** 10.1007/s00066-023-02045-1

**Published:** 2023-02-03

**Authors:** Felix Mehrhof, Paula Bergengruen, Jin-Hong Gerds-Li, Andrea Jahn, Anne Kathrin Kluge, Abdul Parwani, Daniel Zips, Leif-Hendrik Boldt, Felix Schönrath

**Affiliations:** 1 Department for Radiation Oncology, Charité—University Medicine Berlin, Berlin, Germany; 2 Department of Cardiothoracic and Vascular Surgery, German Heart Institute Berlin, Berlin, Germany; 3 Department for Cardiology, Charité—University Medicine Berlin, Berlin, Germany; 4 DZHK (German Center for Cardiovascular Research) Partnersite Berlin, Berlin, Germany; 5 Charité—University Medicine Berlin, Berlin, Germany

**Keywords:** STAR, Cardiac SBRT, LVAD, Heart rhythm, VT

## Abstract

**Purpose:**

Cardiac radioablation (cRA) using a stereotactic single-session radioablative approach has recently been described as a possible treatment option for patients with otherwise untreatable recurrent ventricular tachycardia (VT). There is very limited experience in cRA for patients undergoing left ventricular assist device (LVAD) therapy. We present clinical experiences of two patients treated with cRA for incessant VT under long-term LVAD therapy.

**Methods:**

Two male patients (54 and 61 years old) with terminal heart failure under LVAD therapy (both patients for 8 years) showed incessant VT despite extensive antiarrhythmic drug therapy and repeated catheter ablation. cRA with a single dose of 25 Gy was applied as a last resort strategy under compassionate use in both patients following an electroanatomical mapping procedure.

**Results:**

Both patients displayed ongoing VT during and after the cRA procedure. Repeated attempts at post-procedural rhythm conversion failed in both patients; however, one patient was hemodynamically stabilized and could be discharged home for several months before falling prey to a fatal bleeding complication. The second patient initially stabilized for a few days following cRA before renewed acceleration of running VT required bilateral ablation of the stellate ganglion; the patient died 50 days later. No immediate side effects of cRA were detected in either patient.

**Conclusion:**

cRA might serve as a last resort strategy for patients with terminal heart failure undergoing LVAD therapy and displaying incessant VT. Intermediate- and long-term outcomes of these seriously ill patients often remain poor; therefore, best supportive care strategies should also be evaluated as long as no clear beneficial effects of cRA procedures can be shown. For patients treated with cRA under running ventricular rhythm abnormality, strategies for post-procedural generation of stabilized rhythm have to be established.

## Introduction

In patients with advanced and end-stage heart failure, repeated occurrence of ventricular tachycardia (VT) is one of the hallmarks of disease progression and is commonly treated by implantable cardioverter defibrillators (ICD), catheter ablation, or antiarrhythmic drug therapy (AADT) [1]. Frequently, a combination of these therapeutic concepts is necessary. Patients with symptomatic heart failure despite optimal medical and device-based therapy might be considered for long-term mechanical circulatory support systems including left ventricular assist device (LVAD) on their way towards heart transplantation or as destination therapy with the aim of increased quality of life [2]. Following LVAD implantation, ventricular arrhythmias remain abundant with rates up to 50%, despite successful hemodynamic ventricular unloading. The predominant mechanism of VT post LVAD implantation is in most cases related to a preexisting scar, while only 20% can be attributed to cannula insertion [3]. Especially for patients with mechanical support system as a destination therapy, post-implantation ventricular arrhythmia is an independent predictor of mortality [4].

Cardiac radioablation/radiosurgery (cRA) has recently been reported as a last resort strategy for patients with ventricular tachycardia (VT) unsuitable for catheter ablation (CA) or following unsuccessful procedures [5, 6]. Several case reports and patient series have been published reporting feasibility and early clinical results of this novel approach [7–10]. Currently, several prospective clinical trials aimed at investigation of short- and long-term morbidity and procedure-related side effects as well as treatment efficacy have been implemented. In Europe, the STOPSTORM consortium aims to collect data regarding efficacy and safety in a large and broad VT patient population treated by multiple centers on various treatment platforms [11]. In many of these studies, patients with LVAD are excluded. Others, like the RAVENTA trial [12], do not explicitly rule out patients with LVAD but refer to the occurrence of imaging artefacts which hinder exact target volume delineation. The clinical experience of cRA in patients with LVAD is therefore limited.

In the current report, we describe our experience with cardiac radioablation procedures for two patients bearing LVAD and presenting with persistent VT.

## Case descriptions

### Patient I

Patient I was a 54-year-old male with a long history of non-ischemic dilative cardiomyopathy following chronic excessive alcohol consumption. The patient received an automated implanted cardioverter defibrillator (AICD; Atlas, St. Jude Medical, St. Paul, MN, USA) initially in 2006 following occurrence of repeated ventricular tachycardia episodes. Heart failure was further aggravated following ST-segment elevation myocardial infarction due to thromboembolic occlusion of the right coronary artery in 2011. The patient underwent implantation of a left ventricular assist device (LVAD; HeartMate-II) in 2012. In 2019, he experienced repeated (> 10) ICD shock therapies in the context of an electric storm episode and demanded deactivation of the ICD device thereafter. Due to repeated ventricular fibrillation episodes, the patient received extensive antiarrhythmic medical therapy and underwent multiple catheter ablation procedures in the years 2017 and 2019. In January 2020, the highly symptomatic patient (vertigo, dizziness) was again hospitalized, and repeated VT episodes were treated by external electric cardioversion; a final relapse could only be managed by pharmacological deceleration using high dosages of amiodarone and class Ic antiarrhythmic drugs. A permanent slow VT with a rate of 110–120/min was tolerated due to the functioning LVAD device with a cardiac output of 4.5–5 L/min. With respect to his clinical condition, clinical as well as serological signs of right heart failure were evident, being in line with noninvasive imaging (transthoracic echocardiography showing moderately impaired right heart function and severe tricuspid regurgitation). Even under rate control, symptoms were barely manageable. Coronary angiogram did not reveal any new stenosis.

**Fig. 1:**
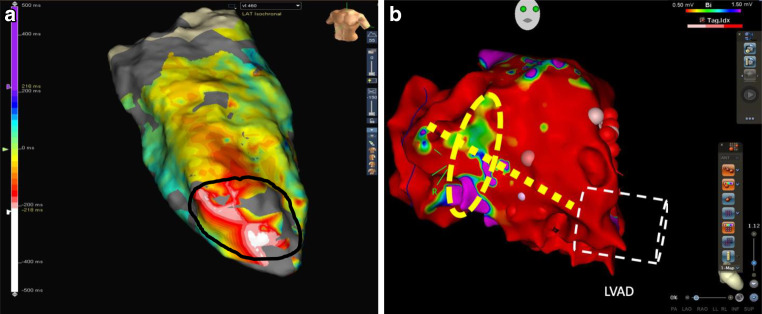
Electroanatomical mapping. **a** Case I: LAO caudal projection of an ultra-high-density activation map (EnSite NavX and Advisor HD-grid, Abbott Medical, MN, USA) of sustained ventricular tachycardia displaying earliest activation (*white spots*) at the anterior apex of the left ventricle close to the insertion of the LVAD cannula. *Black line* describes the interdisciplinarily specified target area. **b** Case II: RAO projection of an ultra-high-density voltage map (Carto 3, Biosense Webster, Diamond Bar, Ca, USA) with diffuse left ventricular low-voltage areas (*red color*). White lines delineate LVAD cannula; the *yellow dashed circle* indicates the possible target area, the *yellow dashed line* depicts the area of last failed catheter ablation

In February 2020, the patient underwent yet another high-density electroanatomic voltage mapping (EAM) procedure (Advisor HD grid^TM^, Abbott Medical, MN, USA), demonstrating a large scar area in the anteroapical and anterolateral region of the massively enlarged left ventricle. Activation mapping with a mapping system (Abbott Medical Devices) showed early activation of ventricular tachycardia near the entrance of the LVAD cannula into the apex of the left ventricle (Fig. 1a). Attempted substrate modification by radiofrequency ablation in this area could not permanently establish a stable sinus rhythm. The patient remained in a highly fragile state with permanent slow VT under ongoing LVAD support.

### Patient II

Patient II was a 61-year-old male with a longstanding history of ischemic heart failure following posterior wall myocardial infarction in 2002. The patient received coronary artery bypass graft surgery in 2012 and was equipped with a LVAD (HVAD HeartWare) due to advanced heart failure symptoms in 2013. The patient had received a two-chamber ICD (Biotronik, Lumax 540 DR-T) in 2010 following repeated ventricular tachycardia. Since 2020 the patient had experienced repeated episodes of ventricular tachycardia, repeatedly terminated by electrical cardioversion and followed by intensified medical antiarrhythmic drug therapy (AADT). In July 2021, an electrical storm episode led to five consecutive shock therapies, demanding a further increase of AADT. Clinically severe heart failure symptoms NYHA class III accompanied by end-organ failure was evident. Coronary angiogram did not reveal any new stenosis. An electroanatomical mapping procedure (CARTO 3; Biosense Webster Inc., Diamond Bar, CA, USA) revealed low-voltage-areas in the inferolateral areas of the left ventricle, reaching towards the LVAD cannula insertion (Fig. 1b). A catheter ablation procedure in the inferolateral area remained without success regarding rhythm restoration; in fact, following the ablation procedure, a change in VT morphology was registered. The patient remained under constant slow ventricular tachycardia tolerated under LVAD-therapy, with a cardiac output of 4.5–5 L/min.

Patient characteristics and clinical details of both patients are outlined in Table 1.

**Table 1:** Patient characteristics and clinical details

	Patient I	Patient II
	Clinical details	
Age at time of treatment, years (t. o. t.)	54	61
Cause of cardiomyopathy	Non-ischemic CM	Ischemic CM
NYHA state/AHA-ACC	IV/D	IV/D
Years since LVAD implantation	8	8
Former catheter ablations	5 (no epicardial)	3 (no epicardial)
Heart rate at t. o. t.	≈ 110/min	≈ 130/min
Antiarrhythmic drugs at t. o. t.	Beta-blocker, amiodarone, flecainide	Beta-blocker, amiodarone

*t. o. t.* time of treatment, *CM* cardiomyopathy, *NYHA* New York Heart Association classification of heart failure, *AHA/ACC* American Heart Association/American college of Cardiology stage of heart failure

### Target volume definition, treatment planning, and stereotactic radiosurgery

In patients I and II, conventional treatment approaches remained unsuccessful; therefore, both patients were evaluated for cRA as a last resort strategy under compassionate use, received extensive patient information from cardiologists as well as radiation oncologists, and gave informed consent. Patients underwent 4D computed tomography (CT) for treatment planning performed with a Somatom Sensation Open CT scanner (Siemens^TM^) as well as a contrast-enhanced cardiac CT (SOMATOM Definition Flash, Siemens^TM^) for coregistration with the planning CT and visualization of the ventricular walls. All available information from the electroanatomical mapping procedures was evaluated in an interdisciplinary setting involving cardiological electrophysiology specialists, radiation oncologists, and medical physicists. For each case, the respective target area was manually transferred to the planning CT and remodeled as a 3D structure including the myocardial wall of the fused contrast-enhanced cardiac CT to result in the gross target volume (GTV). An additional margin resulted in the internal target volume (ITV), accounting for residual cardiac motion as evaluated by coronary excursion in the contrast-enhanced cardiac CT. An additional safety margin was added to the ITV region, resulting in the planning target volume (PTV) which accounts for any residual uncertainties in patient setup and motion. A single dose of 25 Gy with a coverage of 95% (PTV V_25Gy_ ≥ 95%), the near-maximum dose limited to 32.5 Gy (PTV D_2%_ ≤ 32.5 Gy), and recommended peak doses within the ITV above 30 Gy was prescribed. Prescription and reporting for both treatment cases were based on the International Commission on Radiation Units and Measurements (ICRU) report 91 for stereotactic treatments with small photon beams [13]. Treatment was accomplished according to standardized treatment quality requirements for stereotactic radiosurgery [14], with the ICD completely blocked from irradiation and the LVAD directionally blocked from beam interaction. For both patients, treatment was consented by the institutional ethics committee, the patients gave written informed consent after sufficient respite.

#### Patient I

**Fig. 2:**
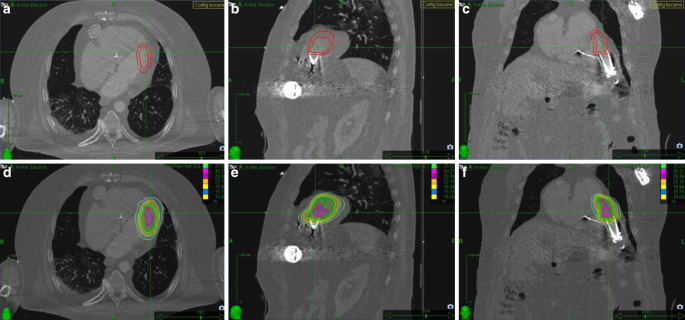
Case I: clinical and planning target volumes in axial (**a**), sagittal (**b**) and coronal plane (**c**) and dose distribution of radiosurgery treatment plan in corresponding levels of planning computed tomography in axial (**d**), sagittal (**e**), and coronal views (**f**). Dose distribution is displayed to the 15 Gy isodose. The *green line* displays the 25 Gy isodose

For patient I, the ITV was enlarged by a 5-mm PTV margin (Fig. 2a–c). Treatment plan generation and dose calculation were done with the Precision^TM^ (version 2.0.1.1) treatment planning system (Accuray Incorporated, Sunnyvale, CA, USA) using the RayTracing dose calculation algorithm (Fig. 2d–f). Treatment was performed with a CyberKnife VSI^TM^ (Accuray Inc., Sunnyvale, CA, USA) using the delineated ICD right ventricular probe tip as a fiducial marker for respiratory motion compensation (Synchrony^TM^ Respiratory Tracking). A total of 117 noncoplanar beams with photon energy 6 MV and IRIS collimation were delivered. Figure 2 shows the planning volumes and dose distribution of the respective treatment plan.

#### Patient II

**Fig. 3:**
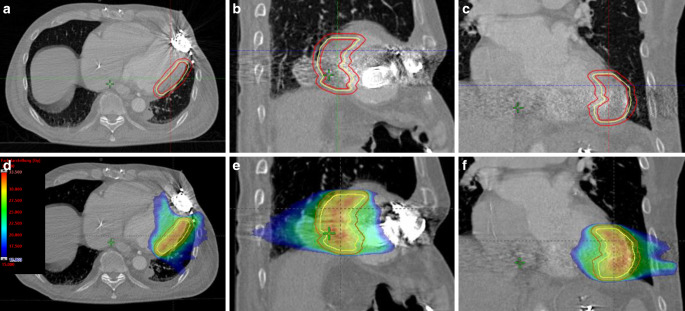
Case II: clinical (*inner red contour*) and planning target volumes (*larger red contour*) and integrated target volume (*yellow line*) in axial, sagittal, and coronal views (**a**–**c**). Dose distribution of radiosurgery treatment plan in corresponding levels of planning computed tomography (**d**–**f**) together with a dose scale (4d). Dose distribution is displayed to 15 Gy; ITV and PTV contours are displayed

For patient II, a Varian True Beam^TM^ STX was chosen for treatment in order to account for the reduced cardiopulmonary condition and avoid longer treatment times. The prespecified ITV was enlarged by 4 mm to result in the PTV (Fig. 3a–c). The treatment plan was created with the Varian Eclipse^TM^ treatment planning system (Varian Medical Systems Inc; Palo Alto, CA; version 15.5) and consisted of four noncoplanar volumetric modulated arcs using 6‑MV flattening filter-free photon beams. The dose distribution was calculated using the AAA/Acuros AXB algorithm (Fig. 3d–f).

LVAD cannula artefacts with disturbances close to the target area complicated treatment planning; however, the treatment technique with high numbers of irradiation directions and the directional blocking of the metal inserts still allows for accurate dose calculation. Table 2 provides some details of the target volumes and treatment and dosimetric specifications for the two cases. Dosimetric guidelines and dose constraints for organs at risk were adopted from the protocol of the German multicenter multiplatform RAVENTA trial [12]. During the treatment period, patients were visually monitored with a camera to continuously observe LVAD function and O_2_ saturation with a pulse oximeter. A cardiotechnician was present during the entire treatment in the control room to ensure the possibility of immediate specialized patient care at any time.

**Table 2:** Treatment details

	Patient I	Patient II
	Treatment and dosimetric details	
Treatment technique	Robotic SBRT	C‑arm linac SBRT
Motion management	Tracking	ITV
ITV volume (ml)	34	78.7
PTV volume (ml)	75.2	134.6
Treatment time (min)	75	25
Monitor units	14,067	15,431
Maximum dose	31.57 Gy	31.41 Gy
Coverage	95.96%	100%

*ITV* internal target volume, *PTV* planning target volume

### Follow-up

The course of treatment was uncomplicated for both patients; neither described any immediate treatment-related reaction or side effects. ICD and LVAD function were controlled immediately following radioablation and showed no signs of dysfunction. Patients were transferred to the cardiologic care unit for further monitoring immediately following treatment. As mentioned above, both patients had to be treated during ongoing VT, with, however, stable circulatory conditions under LVAD treatment with a cardiac output around 4.5 to 5 L/min. No acute side effects were registered in the first 3 days following radioablation.

#### Patient I

Patient I remained stable for the next 8 weeks under intensive care conditions, with an ongoing slow VT around 95/min. AADT was continued unchanged, and two attempts at external cardioversion after 2 and 6 weeks remained unsuccessful. Due to a slightly reduced frequency of the VT, the patient could be stabilized and mobilized to leave the bed. The patient could be discharged home with specialized care 10 weeks following the radioablation procedure and remained mobile with a wheeled walker for several months. However, 5 months following radioablation, the patient was rehospitalized for progressive anemia unrelated to the radioablation procedure; he finally died in multiorgan failure 5.5 months after the radioablative intervention. No further deterioration of arrhythmias was reported.

#### Patient II

Patient II displayed a complex clinical course following radioablation, with initially persisting but slightly decelerated VT. Heart rate decreased from around 190 beats per minute (bpm) to around 130 bpm within the first 6 days after radioablation, with a further decrease to 100–110 bpm within the following 14 days after ablation. Initially, AADT could be carefully reduced; a first attempt at external cardioversion 2 weeks after radioablation remained unsuccessful. Three weeks following cRA, the ongoing VT again accelerated and reached a rate around 140 bpm. An attempt at bilateral stellate ganglion blockage was undertaken with a short-term benefit; the patient could be stimulated and the heart rate controlled by a pacemaker for several days before the VT again accelerated with subsiding effect of the blockage. Finally, the patient deceased 50 days following the radioablation procedure due to progressive end-organ failure.

## Discussion

Ventricular arrhythmias are a common problem in patients bearing a LVAD and are associated with significant morbidity from repeated ICD shocks and with progressive failure of the mostly unsupported right ventricle [15–17]. Catheter ablation in these patients is especially complicated due to technical and procedural challenges such as cumbersome catheter control or catheter entrapment due to altered ventricular filling or interference with the electroanatomical mapping system.

Cardiac radioablation is a novel technique for treatment of therapy-refractory ventricular tachycardia which has been described in a limited and assessable number of case reports and case series. Currently, cRA finds its justification and application mainly in situations when the standard treatment algorithm using antiarrhythmic medication and catheter ablation have failed or seem inappropriate. This might be secondary to drug intolerability to or toxicity of medical therapy, or in cases where catheter ablation procedures have failed or cannot be performed due to technical reasons. In fact, to date, there has only been one completed prospective phase I/II trial reporting results from 19 patients undergoing cRA [18]. In this trial (NCT02919618) in adults with treatment-refractory episodes of VT or cardiomyopathy related to premature ventricular contractions, the participation of patients bearing an LVAD system was excluded per protocol.

The same holds true for another case series published by Lee and colleagues [19]: in this trial, patient inclusion and exclusion criteria were adopted from the abovementioned protocol, and therefore did not include patients bearing a mechanical assist device.

In a retrospective single-center case series of patients with a history of recurrent scar-related VT bearing an ICD, Neuwirth and colleagues report long-term results of these patients who underwent cRA on a CyberKnife platform [20]. In this series, subjects bearing a mechanical assist device were once again excluded from participation.

Another published patient series based on a prespecified study protocol (NCT02661048) investigated clinical outcomes of patients enrolled in the CardioPlan software feasibility study. Feasibility of cRA for the treatment of refractory scar-related VT is reported for a number of 6 patients [21], among them no candidates under LVAD support.

There have been few reports of patient series including subjects with a mechanical assist device. The study by Lloyd and colleagues [22] included a total of 10 patients who 1) failed on at least two antiarrhythmic drugs, 2) additionally failed on at least one radiofrequency ablation, and 3) failed one adjunctive therapy such as mechanical support or sympathetic blockage. Recently, long-term results of this monocentric series were published and reported a total of 14 patients—among them three LVAD patients (one female and two male patients) [23]. All patients had been treated under compassionate use and were reported on in a retrospective manner. All three LVAD patients showed further, nonclinical VT episodes with different morphologies following cRA and underwent cardiac transplantation in the further cause of treatment. Notably, the origin of clinical/remaining VT was localized to the area around the LV cannula.

The report of the preliminary results of the Italian TRA-MI-VT trial [24] (NCT04066517) describes yet another patient bearing a mitroaortic mechanical prothesis and a (not further specified) cardiac support device. Following treatment, the patient completed the 6‑ and 12-month clinical follow-up examinations; a significant reduction in VT episodes could be registered over time and AADT could be reduced after 3 months.

The German RAVENTA trial (NCT03867747) is yet another prospective multicenter, multiplatform trial investigating the safety profile and efficacy of cardiac radioablation for ventricular tachycardia [12]. In this trial, patients with cardiac assist devices are not automatically excluded from the study treatment as long as device-induced image artefacts cause no relevant impairment of target volume definition.

Treatment of patients with ventricular assist devices bears a number of challenges for cRA procedures. Target volume identification critically relies on invasive electroanatomical mapping procedures. This process might be impaired in subjects bearing a LVAD due to locally reduced catheter contact to the endocardium and therefore incomplete mapping. In addition, the treatment planning procedure is based on dedicated computed tomography imaging [25] to ensure analysis of cardiac and respiratory motion and also organ at risk definition. Different strategies have been investigated to optimize treatment accuracy despite cardiac and respiratory motion, among them abdominal compression, respiratory gating, and a tracking technique of the ICD electrode mainly in robotic radiosurgery treatment attempts [26]. Accurate and standardized transformation of the target volume as defined during EAM to the planning CT seems to be of critical importance for successful treatment [27] and might be facilitated by additional software tools [28]. Metal-related artefacts may hamper these efforts in patients bearing a LVAD and therefore menace treatment success. cRA has been shown to be feasible with robotic arm and C‑arm LINAC systems with comparable plan quality [29]; treatment planning depends on dose specification and platform capabilities, with consequences for the method of motion management.

In the patients described in our case reports, rhythm restoration remained unachieved also at later timepoints. For the first patient, a second attempt at external cardioversion 6 weeks following cRA did not result in a stable sinus rhythm. The second patient had to undergo yet another invasive procedure (bilateral blockage of stellate ganglion) when the hemodynamic situation deteriorated after initial stabilization. However, the patient could not be stabilized for a longer period and died with progressive end-organ failure.

Currently, the biological effects of cRA are not completely understood. While animal models indicate the generation of fibrosis and scars in previously damaged myocardial areas following higher radiation doses [30], recent findings indicate yet another mechanism of action involving Notch-dependent radiation-induced reprogramming of cardiac conduction, mainly connexin 43 and Na_V_1.5, leading to fast, yet not immediate changes in rhythm abnormalities [31]. As the radioablation itself does not cause termination of VT, external electric cardioversion is usually applied to restore sinus rhythm or, alternatively, a pacemaker-controlled heart rhythm. The ideal timepoint for post-procedural rhythm normalization by electrical cardioversion is therefore speculative. In the two cases described here, we chose 7 and 14 days post-cRA, respectively, for the initial attempt at rhythm restoration; however, this was without success in both described cases. To the best of our knowledge, there have been no reports of cRA procedures during running slow VT. Few case reports by Scholz and colleagues [32], Jumeau and colleagues [33], and also van der Ree and colleagues [34] describe rescue procedures for patients in repeated electrical storm. However, at the time of the cRA procedure, a normal heart rhythm could be established, and successful retention of normalized heart rhythm was reported for all cases for a period of up to 4 months with only very few episodes of non-sustained VT. In both patients described in this report, electroanatomical mapping revealed a diffuse picture with various potential target sites in the context of large myocardial scars in extremely enlarged left ventricles. These conditions most likely do not pose the best condition for cRA procedures.

## Conclusion

In patients bearing a permanent LVAD, cRA has rarely been described and bears several procedural difficulties. We present data on treatment planning and uncomplicated performance on two different treatment platforms, without, however, achievement of prolonged rhythm restoration. Relapse of VT might be a problem due to multilocular arrhythmogenic foci and the optimal timepoint for post-procedural rhythm restoration is currently unknown. The procedure should, however, be documented for further elucidation of these problems.

We recommend regular documentation and observation of clinical cases of cRA in patients with LVAD, as planned in the observational cohort of the European STOPSTORM registry in order to gain more knowledge and experience in these challenging situations.
